# Artificial Landmarks for Trusted Localization of Autonomous Vehicles Based on Magnetic Sensors

**DOI:** 10.3390/s19040813

**Published:** 2019-02-16

**Authors:** Tobias Mitterer, Harald Gietler, Lisa-Marie Faller, Hubert Zangl

**Affiliations:** Sensors and Actuators Group, Institute for Smart Systems Technologies, Alpen-Adria Universität, 9020 Klagenfurt, Austria; Harald.Gietler@aau.at (H.G.); Lisa-Marie.Faller@aau.at (L.-M.F.); Hubert.Zangl@aau.at (H.Z.)

**Keywords:** wireless sensor network, magnetic sensor, sensor calibration, sensor authentication

## Abstract

Magnetic sensors provide an advantageous alternative localization method, primarily focusing on localization in surroundings where GPS, other radio frequency-based, as well as optical localization do not work or has severe limitations. Suitable for distances in the meter range, such magnetic localization may in particular be useful as artificial landmarks, e.g., for automatic drift correction. To easily use such artificial landmarks, we propose an integration process based on Transducer Electronic Data Sheets. With this approach, the landmarks can be used by passing autonomous vehicles, e.g., UAVs, for re-orientation and re-calibration. During this process, all necessary information such as data formats, reference coordinates, calibration data, provider of the landmark etc. is made known to the vehicle passing by. Based on the provided so-called meta-information, the vehicle itself can decide whether and how to use the provided sensory information. To provide a certain level of trust in the landmarks and their provided information, the corresponding data sheets are certified using a digital signature.

## 1. Introduction

Localization of autonomous vehicles in general and unmanned aerial vehicles (UAVs) in particular, is a complex topic, which, among other factors, strongly depends on the current environment. Common Radio Frequency (RF) approaches using frequencies in the gigahertz range, such as the global positioning system (GPS), work best in open spaces, but show significant deficiencies in buildings and environments. Here radio waves suffer from reflections due to the environment. Visual approaches, such as presented in [1], depend on having a clear line of sight (LoS) to the surrounding area and have problems if this LoS is obliterated by rain, snow, fog, smoke, condensing moisture and other influences. Thus a robust localization scheme usable in an area with those conditions including a concept to introduce security to this scheme is proposed. This paper investigates the use of near field magnetic sensors working in the low radio frequency domain to determine three degrees of freedom (3DOF) as artificial landmarks for localization. Such a magnetic system provides advantages since it is robust with multi-path propagation interference and the localization can be facilitated without the necessity of vision based on cameras or other optical sensors. These magnetic sensors are developed for the use as artificial landmarks to be used in, e.g., landing platforms as they become inefficient at larger ranges due to the required power. Where such landmarks still have the same advantages over commonly used landmarks, which are based on visual cues, like QR codes [2]. These artificial landmarks are automatically detected and integrated into the Robot Operating System (ROS). The ROS can be employed on a UAV which comes into the vicinity of such a landmark, but also on any other vehicle equipped with the necessary hardware. The sensory data can then be used on the mobile platform to re-calibrate its localization algorithm and other devices necessary for its path planning or operation. The description of the landmark and its properties, such as the employed encoding used for the transmitted data, are stored in form of a Transducer Electronic Data Sheet in the style of the IEEE 1451 standard [3]. An important topic when connecting to any artificial landmark is if the information gained from the landmark is trustworthy. With this aspect in mind, optical landmarks, e.g., QR codes, can be manipulated easily as the information is static. Also RF landmarks, such as the proposed ones, can be manipulated to create ’fake’ landmarks.Therefore it is investigated, how a high level of trust can be established between the UAV and the landmarks. The approach is based on electronic data sheets according to the IEEE 1451 format, which are extended by a security part containing a digital signature. With the signature, the mobile platform can validate if it can trust the localization information gained from the landmark. The electronic data sheet is transmitted to the UAV to enable an automatic configuration of the system. Other localization approaches using landmarks are presented in [2], where artificial landmarks based on photoelectric scanning are introduced and in [4], where a Pedestrian Dead Reckoning (PDR) approach combined with QR code based landmarks is presented. Another vision-based artificial landmark approach, which uses template matching, where multiple templates are searched for in an image to calculate distance to the landmark is discussed in [5]. A sensor fusion-based approach using vision-based artificial landmarks in conjunction with a cheap gps module is described in [6]. Most of the aforementioned approaches have the problem that they need a clear field of vision from landmark to the mobile robotic platform which is not the case with the proposed system. A system which works even in foggy environments as it uses highly reflective landmarks in conjunction with a laser sensor can be found in [7]. This approach still has the problem that no obstacles are allowed between the landmark and the mobile robotic platform which is not the case in the proposed system as it works in the low rf spectrum and can thus penetrate a large range of materials. Pertaining security, as most artificial landmarks are passive objects with no computational power, e.g., QR-Codes or other pictures for vision-based landmarks, the authentication aspect is omitted. This means, an attacker coud just create copies of the landmark and place it at wrong positions and thus misleading the UAV. Ref. [8] gives an example on how authentication in wireless sensor networks can be implemented, where neighbouring nodes mutually authenticate each other using a variant of the HB+ protocol. Another authentication approach, where every message between sensor nodes is verified using hashes created with an Elliptic Curve Diffie Hellmann (ECDH) algorithm is described in [9]. An overview over used security schemes in wireless sensor networks is given by [10]. An example of using public key architectures in wireless sensor networks can be seen in [11]. Optimization of artificial landmark placement is discussed in [12]. Concerning the magnetic sensing, related magnetic sensing principles were shown in [13] where 3-axis magnetic sensor arrays are used, and in [14] a detailed presentation of the considered magnetic sensor is given. A more detailed description of the employed processing and read-out hardware, which is based on a Software Defined Radio (SDR) platform, is presented in [15]. Section 2 gives an overview of TEDS, the architecture of the proposed system and provides more information on the developed magnetic sensors, Section 3 explains details of the TEDS implementation and Section 4 introduces and describes the security aspects. Finally, in Section 5 a conclusion is given.

## 2. Architecture

### 2.1. Transducer Electronic Data Sheet (TEDS)

A TEDS after the IEEE standard [3] has mandatory components of META TEDS, CHANNEL TEDS and CALIBRATION TEDS. In the META TEDS, meta information about the artificial landmark, such as the number of sensors or actuators, is specified. The CHANNEL TEDS holds information specific to each sensor, such as the physical units measured in SI units, the encoding e.g., the number of bits per sensor sample, and uncertainty of the sensor. It is worth noting that variations of those parameters can be recorded. Therefore, detrimental effects such as long term aging can be considered and compensated. Finally, the CALIBRATION TEDS contain the calibration information of the sensor according to the used calibration algorithm. It is typically a matrix of polynomial coefficients with one dimension the number of coefficients and the other one defining the number of sensor channels used in the calibration of one specific channel. For the artificial landmark, a user-defined TEDS has to be added as well, containing the absolute position of the landmark itself given in the used coordinate system of the mobile robot.

### 2.2. Software Architecture

**Figure 1:**
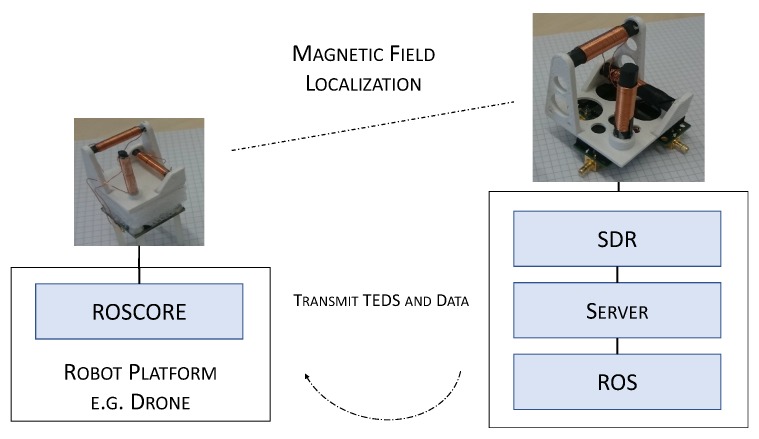
Left is the schematic of the mobile system equipped with a smaller coil setup. Right is the artificial landmark station with a static pose and holds a larger coil system. On the side of the landmark, the coil system is connected to an SDR platform for further signal processing.

The software architecture consists of two components, as can be seen in Figure 1: first the artificial landmark and second the mobile robot platform. The artificial landmark prototype consists of a coil setup made out of three coils, which is connected to the read-out hardware, i.e., the SDR platform, via coaxial cables using SMB plugs. The SDR is connected to a Laptop via a 1 GBit/s Ethernet, this in turn runs software that does the post-processing of the incoming sensor data of the coils. It calculates the position information relative to the artificial landmark. The mobile robot platform drives the transmitting part of the RF based sensor. Depending on the required range, it can be beneficial to use rather low frequencies in order to be robust against reflections possibly shielding objects.

**Figure 2:**
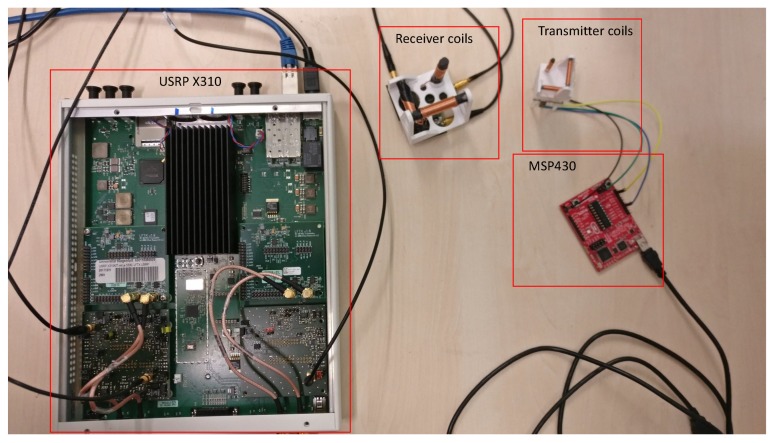
Laboratory prototype of the magnetic sensor. The software defined radio (USRP X310) with a custom daughter board and the receiver coils represent the artficial landmark. Only the smaller transmitter coils need to be on the UAV and the exciatation signals can easily be generated using the controller of the UAV or an additional small microcontroller such as the Texas Instruments MSP430. The equipment is sourced from the institute of the authors, in Klagenfurt, Austria.

In the shown application, a frequency of $f_{c}=457\mathrm{kHz}$ was used, as the basic idea behind the sensing principle stems from avalanche beacons. Additionally, it runs ROS for its own path planning and logic and a Network Capable Application Processor (NCAP) according to [3] specifications with a Wireless Network Processor (WNP). The WNP creates a low power wireless sensor network. As soon as the artificial landmark is in range of the NCAP and WNP, running on the mobile robot platform, it connects to the NCAP. The landmark then identifies itself as a newly connected sensor node and transmits the Transducer Electronic Data Sheet (TEDS) stored on its memory to establish correct interpretation of the incoming data stream and identify which information is provided. After this step the NCAP converts the raw data stream, coming from the artificial landmark, into position information, creates a ROS node for the sensor and sends the data into the ROS system. The absolute position of the landmark, and the uncertainty of the magnetic sensors is stored in its TEDS, and transferred into ROS as a parameter of the created ROS node. The controller running on the mobile robot platform checks available ROS nodes and, as soon as a node classified as position providing artificial landmark is found, it requests the absolute position of the landmark from the ROS parameter server and hooks into the incoming position information data stream coming from the artificial landmark ROS node. From both, the absolute position of the landmark, and the relative position information gained, the controller then calculates its own absolute position respecting the uncertainty of the sensor. The used laboratory prototype can be seen in Figure 2.

### 2.3. Hardware Architecture

**Figure 3:**
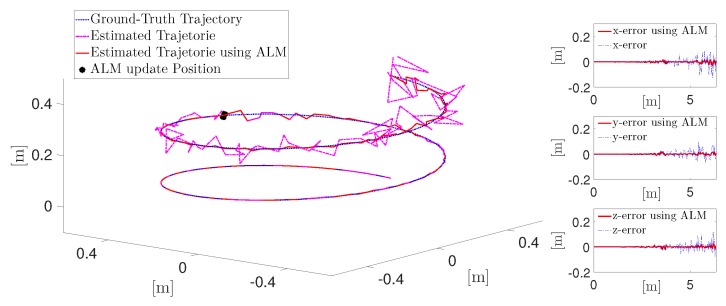
Experimental results for a simulation of UAV flight with drift and drift correction through artificial landmark.

One motivation for these artificial landmarks employing magnetic sensors is that recently studied VIO-based pose estimation schemes drift over time, especially in GPS denied environments. This results in the UAV missing its goal, if set to fly to a position starting at the last well-defined position. Therefore these artificial landmarks are used to counteract the drift in the localization of the mobile platform. In Figure 3, the position marked with a black dot, is where the UAV passes an artificial landmark, which automatically connects to the UAV and supports it with its relative position and the landmark position. Using this information, the UAV calculates its current absolute position, with respect to the uncertainty of the magnetic sensor and adjusts the planned route to reach its goal. Additionally, a possible position drift can stem from a scale error of a visual Simultaneous Localization and Mapping (SLAM) system [16] if the system is equipped with a camera. If only an inertial measurement unit (IMU) is on board, such a drift stems from using the PDR approach as mentioned in [4]. Figure 3 shows a simulation of a flight path via an IMU navigation where the noise is modeled as Arbitrary White Gaussian Noise (AWGN) and the noise and specifications of the analog devices ADIS16448 are used. The flight path is defined by the white noise behavior of the accelerometer, which leads to a second order random walk behavior in the position information [17].

The used magnetic sensor for the artificial landmark is a 3D-printed prototype, consisting of three orthogonally placed magnetic coils, where the relative position of each coil is known. Three smaller and, more important, lighter magnetic coils are placed on the UAV and used as transmitters. The relative position of the UAV with respect to the artificial landmark is estimated by using the received signal strengths. The transmitting frequency is located in the low RF band in order to be robust against reflections and a large class of occluding objects.

## 3. Electronic Data Sheet Approach

The approach is based on the IEEE1451.X Standards and implements an NCAP similar in function to the one described in the standard which is implemented in the python programming language. It therefore conveys the base on which the automatic detection of new sensors builds. An extension to the Standard is that the data sheet describing the artificial landmark is created in the Extensible Markup Language (XML) for better visibility and understanding of each part of the data sheet. This XML file is then parsed into a memory efficient format and written into the flash on the artificial landmark.

The used data sheets IEEE 1451.X TEDS were used because they can be stored in a compressed binary format in a flash memory on the transducer itself, which means no external information is needed to describe the sensor as the whole description is already on the transducer. Most other Data sheet approaches which are commonly used to identify transducers or devices need to be connected to some kind of Master node which has access to a device database and identifies the devices with it. Other data sheet approaches compared in a prerequisite study were SensorML [18], Electronic Device Description Language (EDDL) [19], Semantic Sensor Net Ontology (SSNO) [20], the Wolfram Language [21] and Electronic Data Sheets in CANOpen [22].

## 4. Landmark Validation

When a mobile platform gets the notification that an artificial landmark providing localization information is in its vicinity, it needs to be able to trust the source of the information and the information itself, as they could be fake to maneuver the mobile platform away from the planned destination to another goal. This is not only true for mobile platforms and their localization information but in overall most systems which presently rely on information gained from a remote origin, e.g., a sensor. The ability to trust sensors and their data is a highly discussed topic as they are used in most of today’s life and falsified data can lead to many problems.

**Figure 4:**
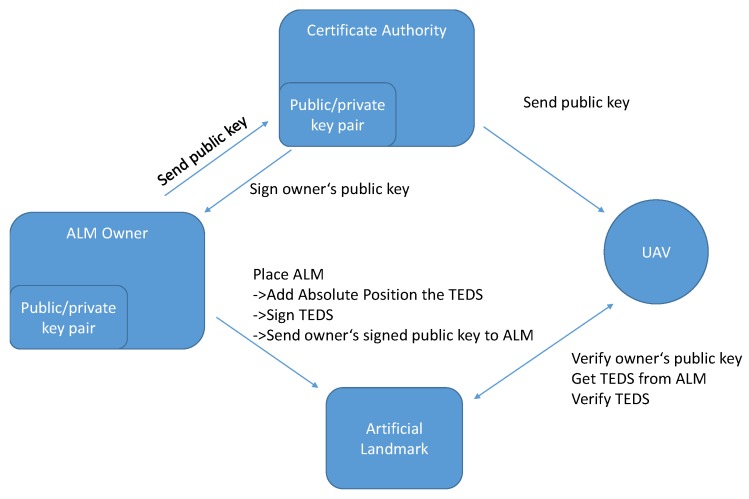
Flow graph of landmark deployment and security process.

The security chain from deployment of the artificial landmark to the connection between landmark and UAV is illustrated in Figure 4. As a root of trust, the certificate authority (CA) signs a public key of a trustworthy instance of a landmark owner or other trusted party and returns the signed key to it. During the actual deployment of such an Artificial Landmark, the landmark owner or other trusted party can then sign the datasheet of the landmark with its own private key after it has added the absolute position of the newly deployed artificial landmark to the TEDS and saves it on the landmark together with its own signed public key. To ensure that an attacker cannot move the artificial landmark, a security process is running in the background on the artificial landmark and monitors information from a locally installed IMU and additionally detects if the landmark is detached from its fixture similar to sensors in an alarm system. If the process detects movement, it will automatically turn off the landmark. This process provides security against illegal movement of the ALM. When an UAV gets close to a landmark, it receives the signed TEDS and the signed public key of the landmark owner from the ALM. With the public key from the CA, which is known by all UAVs, the UAV can validate whether the landmark owner is a trusted party by the CA. Consequently, it is not needed that all trusted landmark owners or deployers (i.e., their public key) need to be known by the UAV. Trustworthiness can be validated at the time when the landmark is encountered. Once the landmark owner is found to be trustworthy, the TEDS can be verified with the public key of the trusted landmark owner. The UAV does not need to contact the landmark owner at any time, which means the landmarks themselves can be owned and deployed by different entities with the single constraint that they can only be trusted if they have been vouched for by the CA.

To provide the above mechanisms, a security extension to the IEEE 1451.X standard is proposed The proposed extensions can be seen in Table 1, where an optional TEDS is described, containing tags for a signature and other relevant information like the algorithm used to create the signature. Additionally to guarantee isolation between the landmark owners and the UAV as explained above, the landmark owner public key and a signature calculated over it using the CA private key is included in the extension.

The most widely used signature algorithms are the Rivest, Shamir and Adleman (RSA) algorithm, the Digital Signature Algorithm (DSA) and the Elliptic Curve Digital Signature Algorithm (ECDSA) [23], and the ElGamal Signature Scheme [24]. Defined in the proposed Security TEDS are these four algorithms for key generation and signature verification, with the possibility for the manufacturer of the artificial landmark to use a custom algorithm and define it in the TEDS. The enumeration for the encryption algorithms can be seen in Table 2 for field 8 and for the used hashing algorithm in Table 3.

The requirements on the signing algorithm in the described application of the mobile platform are that it guarantees authenticity of a landmark under the specification that the private key remains a secret, and that the operation to be computed on the mobile platform is optimized for low computational effort, as a mobile platform is bound by energy constraints. The key generation as well as the generation of the signature itself is done by the manufacturer of the artificial landmark beforehand. The verification of the signature is the only part which needs to be performed on the passing mobile platform.

Of the previously mentioned algorithms, RSA provides better computation times for verification but ECDSA works best for energy constrained devices despite taking a bit longer in the verification process. An example of an optimized ECDSA signing and verification application can be seen in [25].

Using the ECDSA encryption algorithm, the electronic data sheets get a signature which is written into the security TEDS. The keys for signature generation and verification are generated by the same application, which also creates the TEDS and passes the information to the landmark. The public key for verification is also sent to a trusted certificate authority (CA) where a mobile platform can get the key before starting its task or when docked to a landing platform. This way the UAV can be sure to trust the key itself as well as it comes from a trusted source.

**Figure 5:**
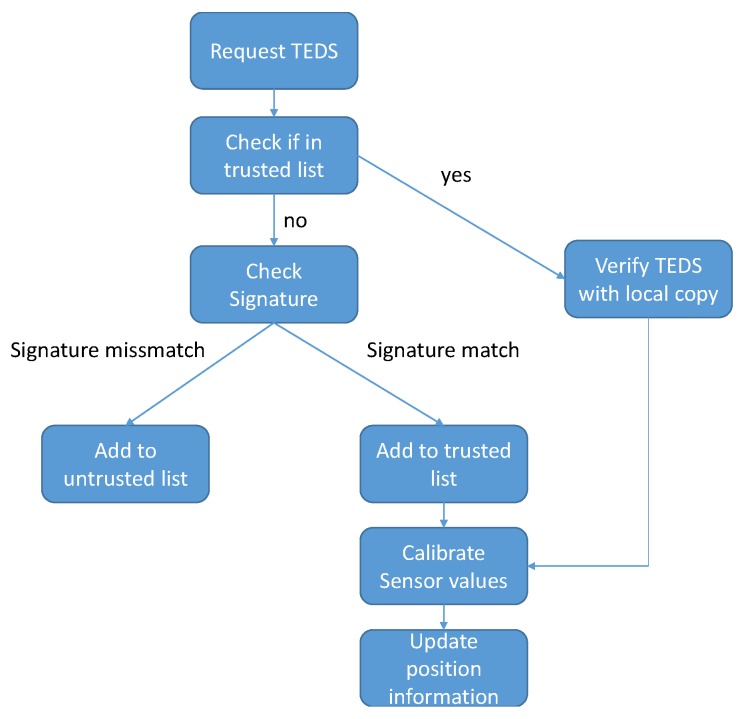
Flow graph of landmark verification process.

The verification process is rather straight forward and can be seen in Figure 5, where first the TEDS are retrieved and as an added step, the signature from the security TEDS is checked against the public key stored in the UAV and, if it checks out, the artificial landmark is added to a local list of trusted landmarks in the UAV by Universally Unique Identifier (UUID). If the mobile platform finds the landmark again in a timeframe given by a timeout value, the verification process is simplified, by only checking the TEDS of the landmark with the local copy of the TEDS of the landmark for differences. After the verification succeeds, the process continues as specified in Section 2.2. If the verification fails, the UUID of the landmark is added to a local list of untrusted artificial landmarks for a given timeframe, to refrain from permanently banning a UUID value if it was spoofed, and the UAV ignores sensor values coming from the landmark. Additionally, as every packet coming from the landmark contains the landmarks UUID, the mobile platform checks the UUID in the packets with the UUID specified by the TEDS to verify that the landmark and its corresponding TEDS correlate.

## 5. Conclusions

In this paper, an approach to automatically connect artificial landmarks, consisting of magnetic sensors, to passing mobile robot platforms, is proposed. The magnetic sensors consist of two parts, with a transmitter on the mobile robot platform and a receiver on the artificial landmark. The authentication and configuration is done via IEEE 1451 TEDS stored on the artificial landmark where an extension to the TEDS in regards to authentication of the landmark to the mobile robot platform is proposed.

## Figures and Tables

**Table 1:** IEEE 1451.0 security TEDS definition.

Bit#	Field	Description	Type	#Octets
-		Length	UInt32	4
0–2	-	Reserved	-	-
3	TEDSID	TEDS identification header	UInt8	4
4	LastModified	Date when Signature was last changed	TimeInstance	8
5	Signature	Signature calculated over TEDS	UInt8	NOTE 1
6	OwnerPK	Public key of Landmark owner	UInt8	NOTE 2
7	SignaturePK	Signature calculated over Public key	UInt8	NOTE 2
8	UsedEncAlg	Encryption Algorithm used	UInt8	1
9	UsedHashAlg	Hashing Algorithm used	UInt8	1
-		Checksum	UInt16	4
NOTE 1—depends on length of TEDS				
NOTE 2—depends on the used algorithms				

**Table 2:** IEEE 1451.0 security TEDS used encryption algorithm enumeration.

Bit#	Algorithm	Description
0	RSA	Rivest, Shamir and Adleman
1	DSA	Digital Signature Algorithm
2	ECDSA	Elliptic Curve Digital Signature Algorithm
3	ElGamal	ElGamal Signature Scheme
4–128	Manufacturer reserved	
129–255	Reserved	

**Table 3:** IEEE 1451.0 security TEDS used hashing algorithm enumeration.

Bit#	Algorithm	Description
0	MD5	Message Digest Algorithm 5
1	SHA-256	Secure Hash Algorithm 2-256
2	SHA-512	Secure Hash Algorithm 2-512
3–128	Manufacturer reserved	
129–255	Reserved	
